# Human DKK1 and human HSP70 fusion DNA vaccine induces an effective anti-tumor efficacy in murine multiple myeloma

**DOI:** 10.18632/oncotarget.23352

**Published:** 2017-12-17

**Authors:** Ting-Ting Liu, Yang Wu, Ting Niu

**Affiliations:** ^1^ Department of Hematology & Research Laboratory of Hematology, West China Hospital, Sichuan University, Chengdu, P.R. China; ^2^ Department of Internal Medicine, No. 4 West China Teaching Hospital, Sichuan University, Chengdu, P.R. China; ^3^ State Key Laboratory of Biotherapy and Cancer Center, West China Hospital, West China Medical School, Sichuan University, Chengdu, P.R. China

**Keywords:** DKK1, hHSP70, fusion genetic vaccine, multiple myeloma, xenogeneic immunity

## Abstract

Dickkopf-1 (DKK1) is an ideal target for the immunotherapy of multiple myeloma. Heat Shock protein70 (HSP70) is a class of important molecular chaperone to promote antigen presentation. Homologous xenogeneic antigens can enhance immunogenicity and induce stronger anti-tumor immune response than that of allogeneic ones. Therefore, we constructed human DKK1 and human HSP70 DNA fusion vaccine (hDKK1-hHSP70), and then determined its anti-tumor immuno- genicity and anti-tumor effects on immunizing BALB/c mice already inoculated with NS-1 murine multiple myeloma cells in prophylactic and therapeutic models using cytotoxic T lymphocytes, enzyme-lined immunosorbent assay, flow cytometry, immunohistochemistry and Hochest staining. The side effects of vaccines were also monitored. We found that hDKK1-hHSP70 fusion vaccine could significantly inhibit tumor growth and prolonged the survival of the mice, whether prophylactic or therapeutic immunotherapy in vivo, by eliciting both humoral and cellular tumor-specific immune responses. A significant decrease of proliferation and increase of apoptosis were also observed in the tumor tissues injected with hDKK1-hHSP70 vaccine. These findings showed the xenogeneic homologous vaccination had stronger immunogenicity and minimal toxicity. Our study may provide an effective and safety immonutheraphy strategy for multiple myeloma.

## INTRODUCTION

Multiple myeloma (MM) is a second prevalent hematopoietic malignancy [1]. In the United States, there are about 20,000 new cases and 10,000 deaths of MM annually [2-4]. MM is still an incurable disease, although the medical conditions are constantly improving [3, 5, 6].

The important cause of treatment failure comes from poor tolerability and drug resistance [3, 7]. Therefore, some novel therapeutic intervention should be developed. Anti-tumor immunotherapy may be a useful and effective treatment for long-lasting control of minimal residual disease (MRD) because of well-tolerated and unlikely cross-resistant with current drugs [3, 6, 8, 9]. Anti-tumor vaccine for prevention and treatment of tumors is one of the significant and valuable research areas in the anti-tumor immunotherapy.

The critical step of preparation of tumor vaccines is to identify tumor specific antigens (TSAs). Dickkopf-1 (DKK1), a secreted antagonist for Wnt signaling pathway, can inhibit osteoblasts and activate osteoclasts. Therefore, it plays an important role in regulating myeloma bone disease (MBD). Almost all MM cells, not normal tissues, express DKK1 specifically [10, 11]. As a result, DKK1 is regarded as one of the TSAs for MM and a potentially important antigenic target for anti-myeloma immunotherapy [11]. Qian et al’ studies showed that active DKK1 vaccination could protect and treat murine multiple myeloma [12].

However, it is key to overcome immune tolerance for effective immunogenicity against cancer. Some experiments have demonstrated that the homologous xenogeneic immunity can enhance immunogenicity and induce stronger anti-tumor immune response because xenogeneic vaccines can effectively break the nature immune tolerance to self-tumor antigens [13-16].

Moreover, the gene adjuvant is considered as an effective way because of enhance immunogenicity of genetic vaccine. Heat shock protein70 (HSP70), especially human HSP70 (hHSP70), is regarded as one of very useful and valuable immune adjuvants because it can induce the maturation of dendritic cells (DCs) and activation of helper T cells (Th1) by combining to CD40 [17-21].

In the present study, we constructed the mice DKK1 (mDKK1), human DKK1 (hDKK1), mDKK1-hHSP70, hDKK1-hHSP70 DNA vaccines. Compared with others vaccine groups, the results showed that hDKK1-hHSP70 DNA fusion vaccine could markedly inhibit tumor growth and prolong the survival against murine multiple myeloma models through inducing humoral and cellular tumor-specific immune responses. The results may provide an effective immonutheraphy strategy for multiple myeloma.

## RESULTS

### Identification of vaccines and western blot assay

**Figure 1:**
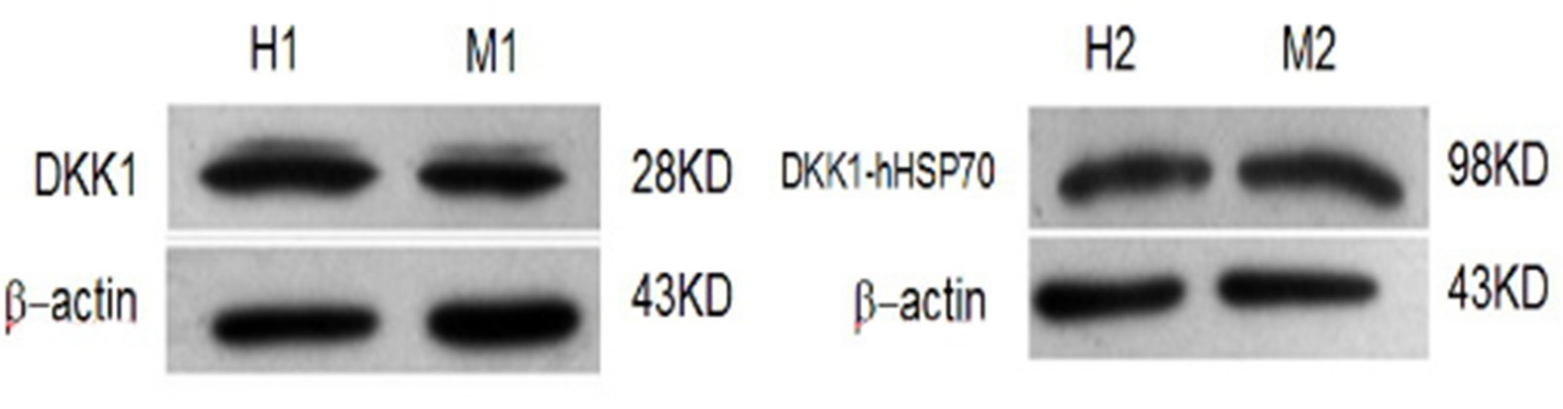
Identification of DKK1 by Western blot H1. The expression of HEK293T cells were transiently transfected with pcDNA3.1-hDKK1; M1. The expression of HEK293T cells were transiently transfected with pcDNA3.1-mDKK1; H2. The expression of HEK293T cells were transiently transfected with pcDNA3.1-hDKK1 -hHSP70; M2. The expression of HEK293T cells were transiently transfected with pcDNA3.1-mDKK1-hHSP70.

**Figure 2:**
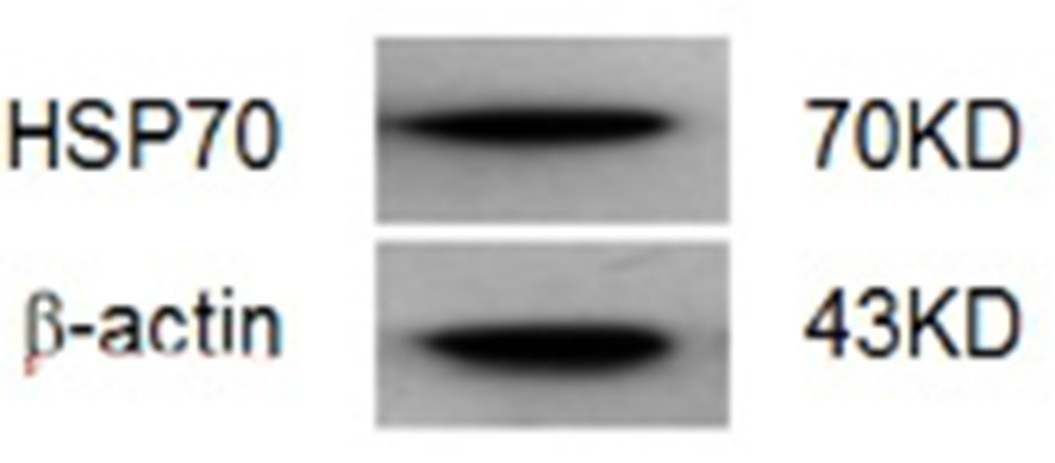
Identification of HSP70 by Western blot

Five recombinant plasmids of pcDNA3.1-mDKK1, pcDNA3.1-hDKK1, pcDNA 3.1-hHSP70, pcDNA3.1-mDKK1-hHSP70, pcDNA3.1-hDKK1-hHSP70, were succe- ssfully constructed as confirmed by diagnostic digestions and DNA sequencing. After transfection of HEK293T cells, Western blot assay showed that pcDNA3.1-mDKK1, pcDNA3.1-hDKK1 could express a 28KD protein band respectively, while pcDNA3.1-mDKK1-hHSP70, pcDNA3.1-hDKK1-hHSP70 respectively express a 98KD protein band that could specifically react with anti-DKK1 antibody (Figure 1). The pcDNA3.1-hHSP70 could expressed a 70KD protein band reacted specifically with anti-HSP70 antibody (Figure 2).

### DKK1 was expressed in NS-1 murine myeloma cells

**Figure 3:**
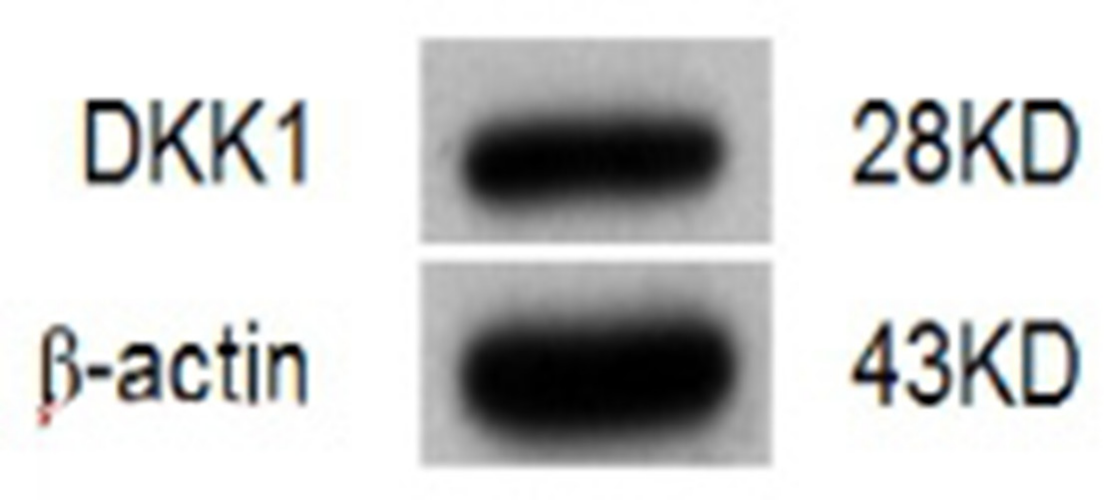
Identification of DKK1 in NS-1 myeloma cells by Western blot

The expression of DKK1 in NS-1 mouse myeloma cells was analyzed by western blot. DKK1 protein was determinated in a 28KD protein band (Figure 3).

### Xenogeneic hDKK1-hHSP70 fusion DNA vaccine significantly suppresses tumor growth of murine multiple myeloma

**Figure 4:**
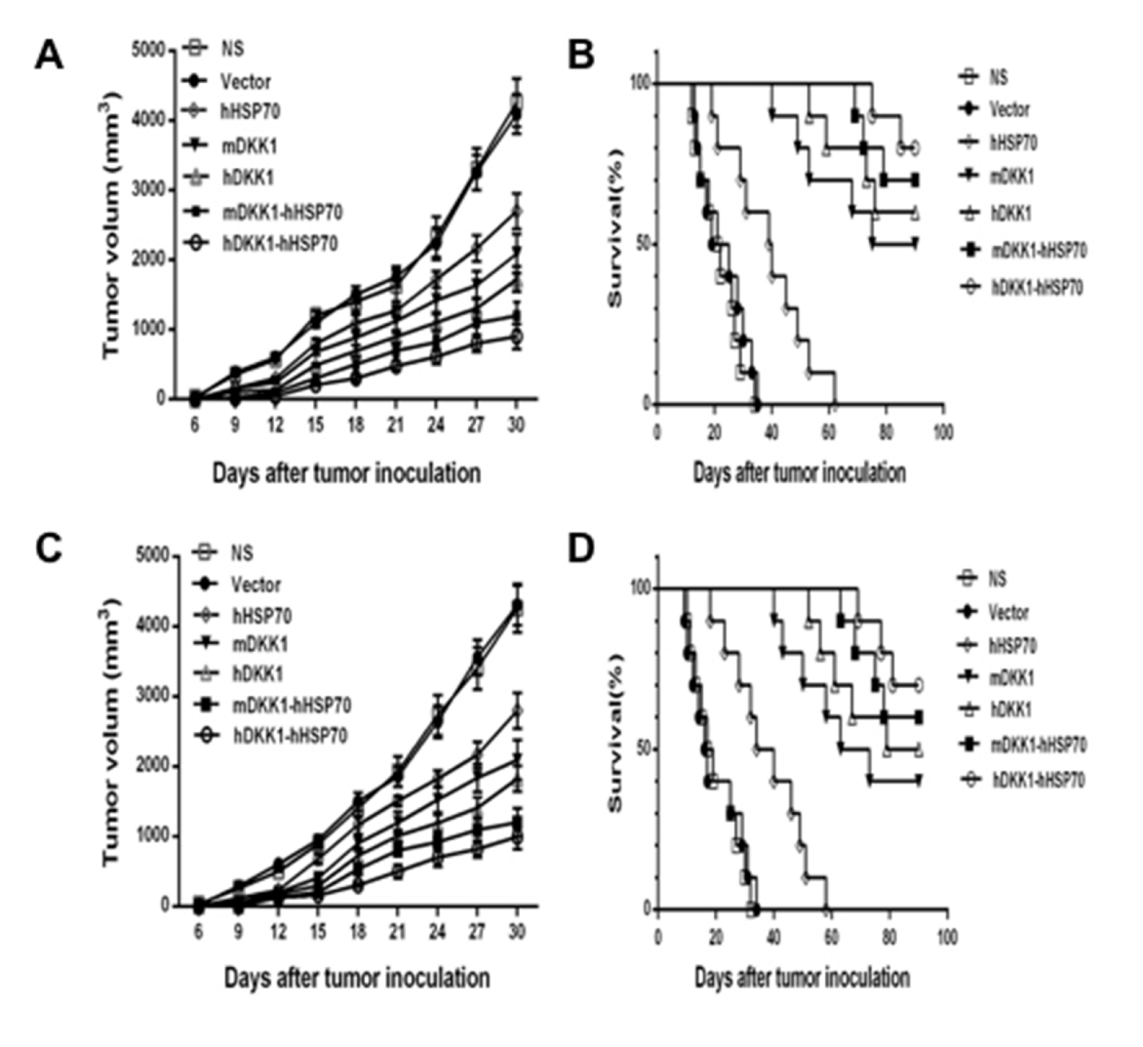
Tumor volumes and survival fractions of tumor-bearing mice **A.** The tumor volume of mice in prophylactic model; **B.** The survival time of tumor-bearing mice in prophylactic model; **C.** The tumor volume of mice in therapeutic model; **D.** The survival of bearing-tumor mice in therapeutic model.

In the prophylactic model (Figure 4A and 4B), mean tumor volume of inoculated NS-1 mouse multiple myeloma was approximately 1423 ± 150 mm^3^ in NS groups and 1501 ± 109 mm^3^ in vector groups on day 18. However, the mean tumor volume was only 397 ± 69 mm3 in the hDKK1-hHSP70 fusion vaccine group (*P* < 0.01 *versus* control), which suggested that the tumor was significantly inhibited in this group. Furthermore, the survival fraction of tumor-bearing mice was significantly prolonged in the hDKK1-hHSP70 group, in which eight of ten mice still survived on day 90 (*P* < 0.05 *versus* control). These results suggested that vaccination of mice with the hDKK1-hHSP70 vaccine inhibited the tumorigenesis and growth of inoculated NS-1 mouse multiple myeloma.

In the therapeutic model (Figure 4C and 4D), by day 21 after NS-1 mouse myeloma cells were inoculated into mice, the mean volumes of in NS group, the Vector group, the HSP70 group, the mDKK1 group, the hDKK1 group, the mDKK1- hHSP70 group, the hDKK1-hHSP70 group were 1926 ± 215 mm3, 1855 ± 157 mm3, 1506 ± 71.5 mm3, 1193 ± 152 mm3, 1008 ± 84.5 mm3, 796 ± 73.4 mm3 and 498 ± 95.2 mm3, respectively. In the NS or vector groups, the growth of tumor was fast, however, it was significantly suppressed in hDKK1-hHSP70 group (*P* < 0.01 *versus* control). Furthermore, seven of ten tumor-bearing mice still survived in hDKK1-hHSP70 fusion DNA vaccine groups on day 90. However, all of the tumor-bearing mice in NS and vector groups died within 40 days.

**Figure 5:**
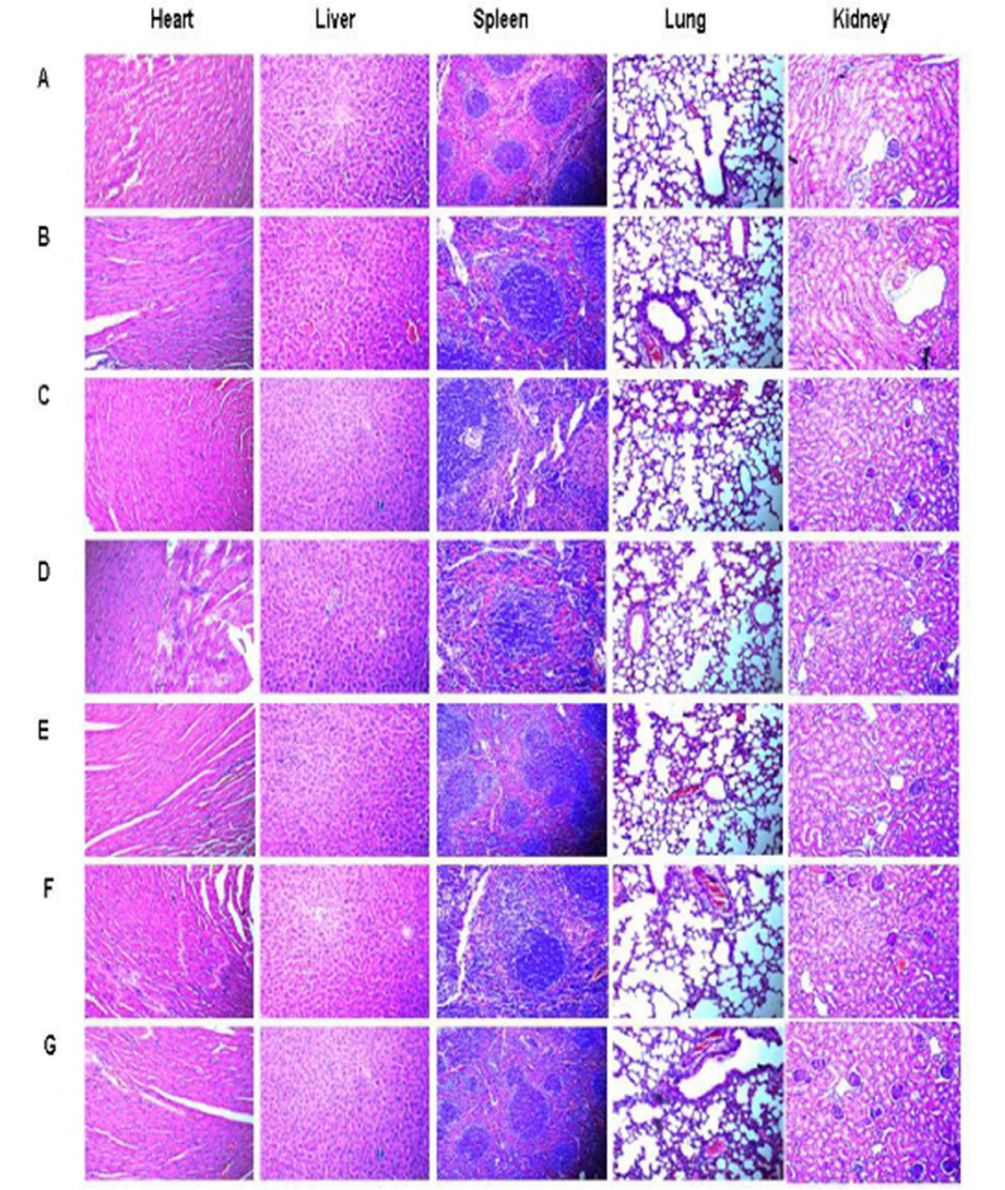
HE staining in tissues of heart, liver, spleen, lung and kidney (×400) **A.** NS; **B.**Vector; **C.** hHSP70; **D.** mDKK1; **E.** hDKK1; **F.** mDKK1-hHSP70; **G.** hDKK1- hHSP70

During the entire immunization process, there was no detectable the toxicity including weight loss, ruffed fur, diarrhea, and anorexia. Hematoxylin-eosin staining (HE staining) was performed in order to assess the the potential toxic effects on internal organs. We haven’t detected obvious pathological changes in the heart, liver, spleen, lung and kidney of the treated mice (Figure 5) by HE staining.

### Cytotoxic T lymphocytes responses plays a critical role in anti-cancer activity of hDKK1-hHSP70 vaccine

**Figure 6:**
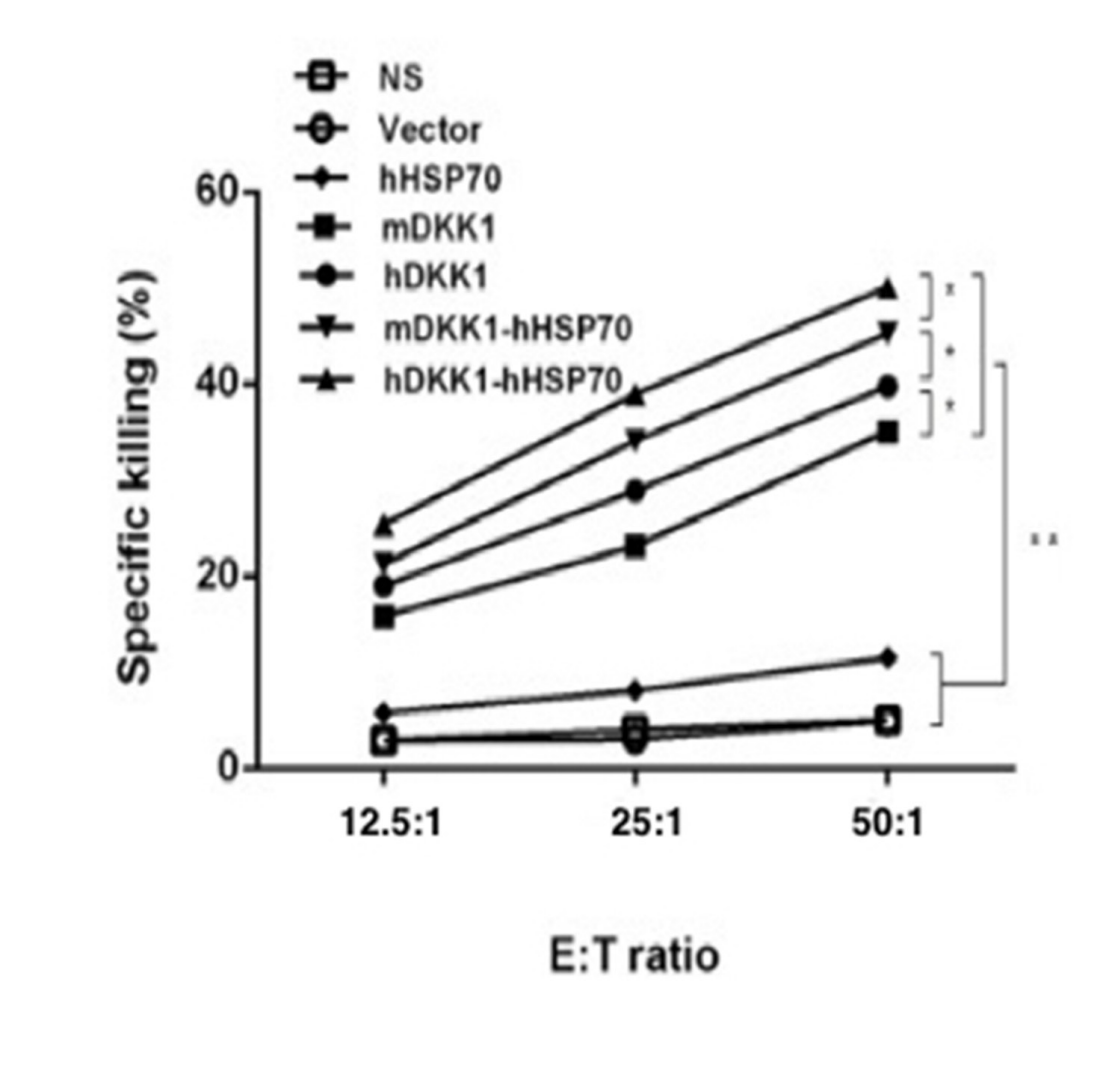
Killing activity of cytotoxic T lymphocyte The cytotoxicity of splenocytes against NS-1 cells was examined by LDH assay. The CTL effect of mice in hDKK1-hHSP70 fusion vaccine was highest among the groups (*P* < 0.05). * *P* < 0.05, ** *P* < 0.01.

CTL activity was carried out using the standards lactate dehydrogenase (LDH) method in order to evaluate the cell-mediated immune response stimulated by the hDKK1-hHSP70 vaccine. The ratio of effector cells: target cells were 12.5:1, 25:1, and 50:1. The effector-to-target (E: T) ratio of 50:1 reached 50.1±1.2% in hDKK1-hHSP70 group. However, there were 45.2±0.9%, 39.74±1.0%, 35.1±0.8%, 11.5±0.6%, 4.9±0.5%, 5.1±0.4% in mDKK1-hHSP70, hDKK1, mDKK1, hHSP70, Vector, NS groups respectively (Figure 6, *P* < 0.01).

### Xenogeneic hDKK1-hHSP70 fusion vaccine increased the frequencies of CD4+ INF-γ+ T cells, CD8+INF-γ+ T cells and decreased the frequencies of CD4+ CD25+FoxP3+ regulatory T cells in spleen

**Figure 7:**
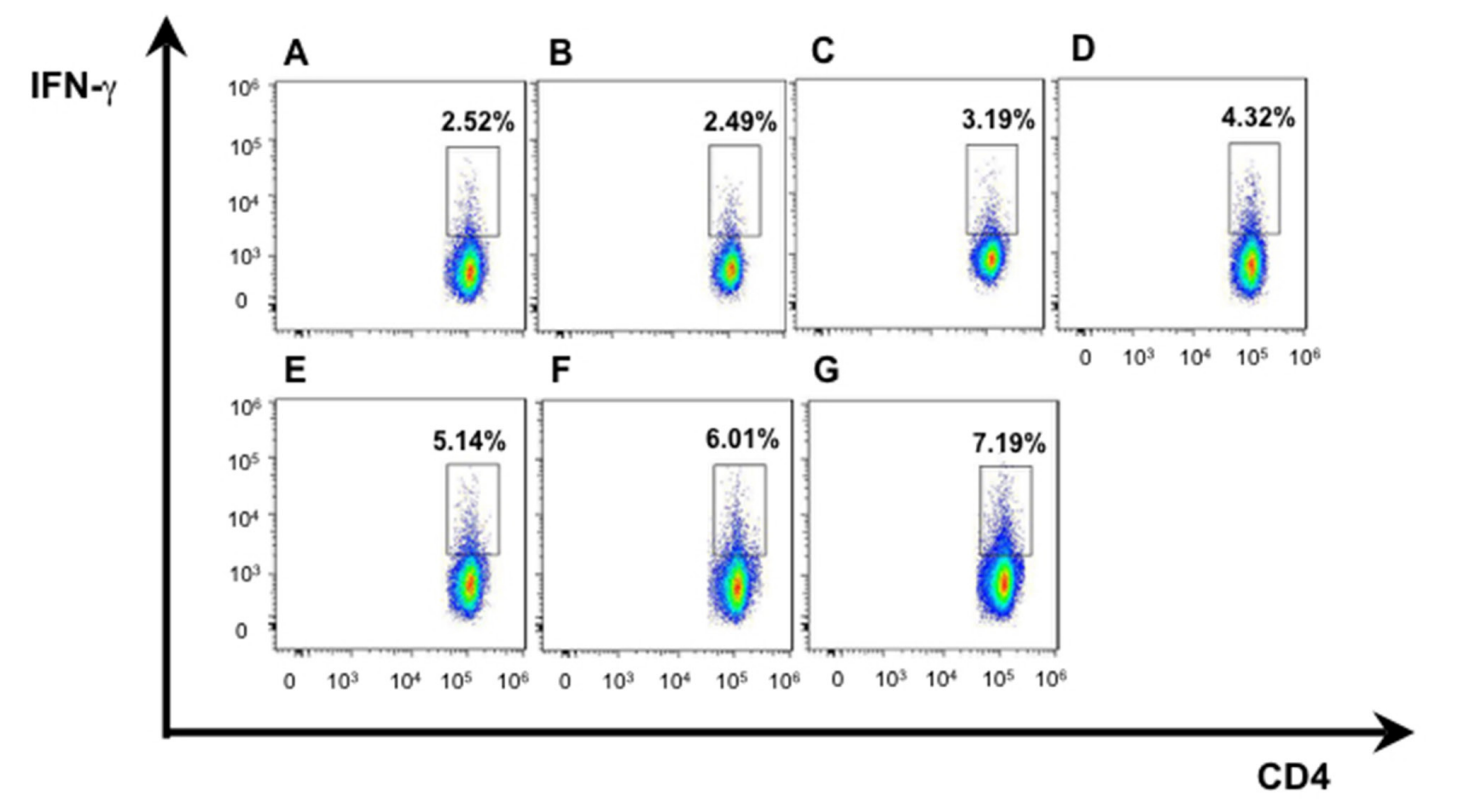
IFN-γ intracellular staining in CD4^+^ T cells by flow cytometry **A.** NS; **B.** Vector; **C.** hHSP70; **D.** mDKK1; **E.** hDKK1; **F.** mDKK1-hHSP70; **G.** hDKK1-hHSP70.

**Figure 8:**
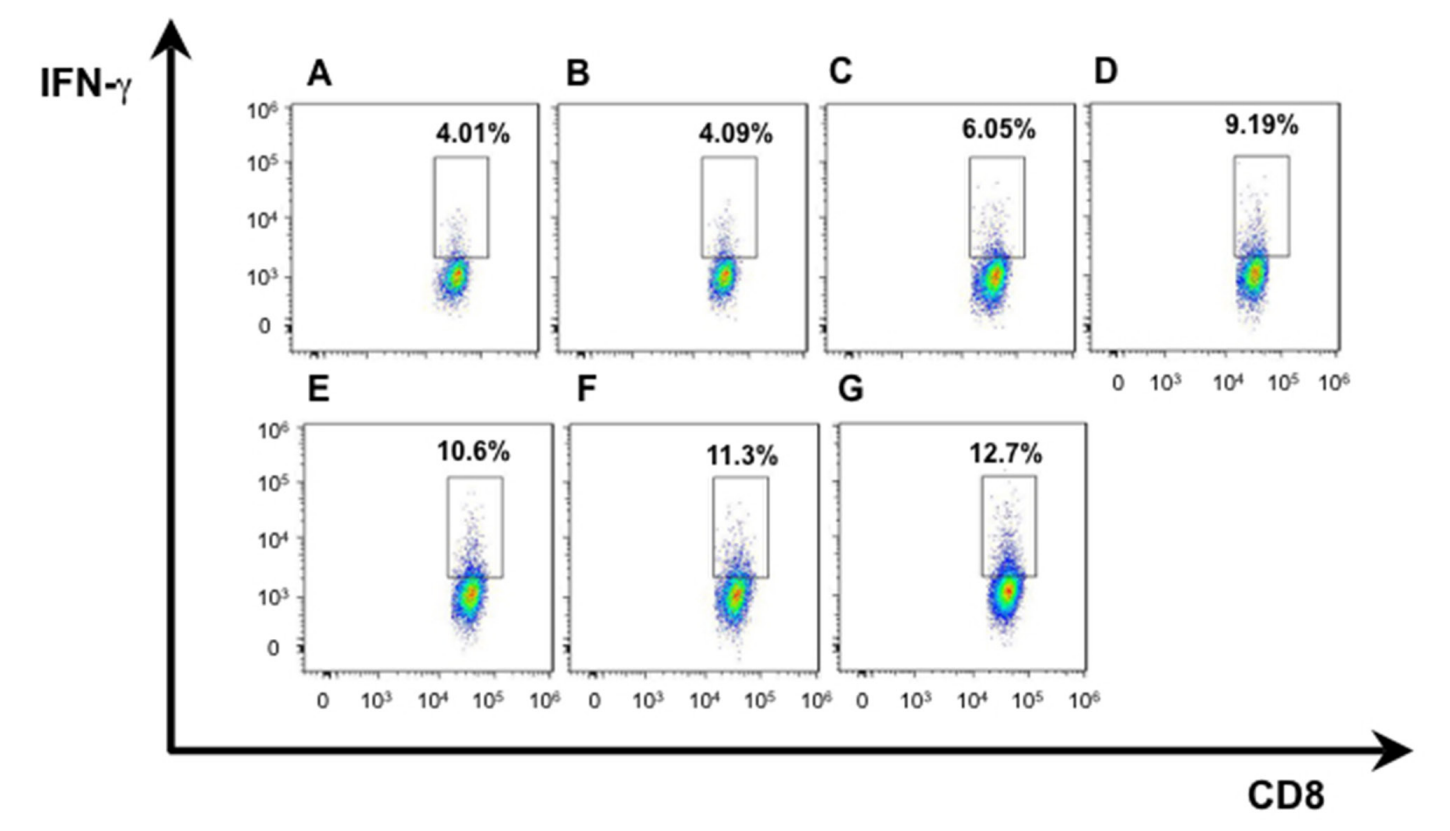
IFN-γ intracellular staining in CD8^+^ T cells by flow cytometry **A.** NS; **B.** Vector; **C.** hHSP70; **D.** mDKK1; **E.** hDKK1; **F.** mDKK1-hHSP70; **G.** hDKK1-hHSP70.

**Figure 9:**
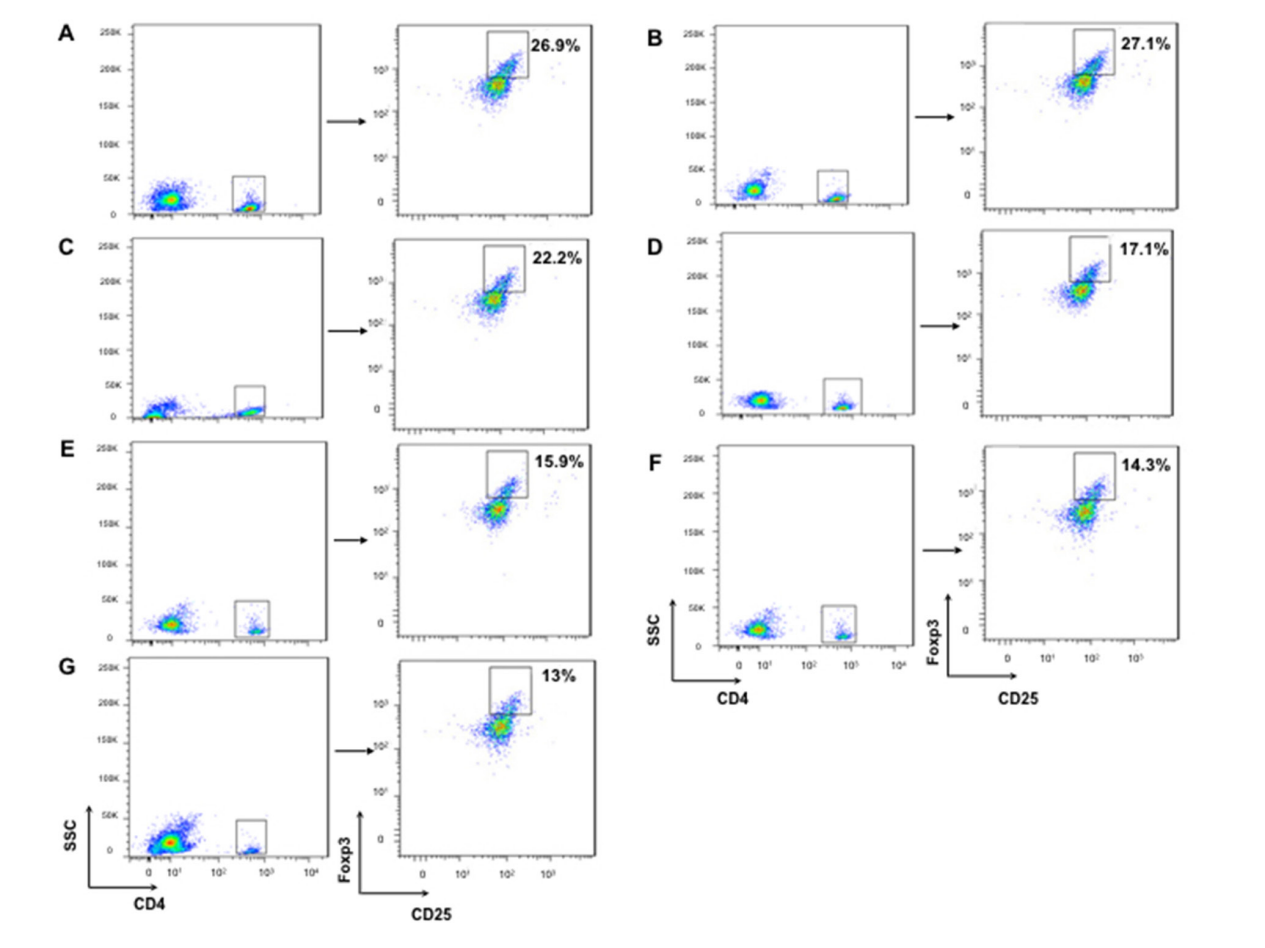
CD4+CD25+Foxp3+ regulatory T cells staining by flow cytometry Firstly, we analyzed the CD4+ cells, and then analyzed the CD25+ and Foxp3+ cells from CD4+ cells. **A.** NS; **B.**Vector; **C.** hHSP70; **D.** mDKK1; **E.** hDKK1; **F.** mDKK1-hHSP70; **G.** hDKK1- hHSP70.

We isolated T lymphocytes and preformed CD4+IFN-γ+ and CD8+ IFN-γ+ double staining for exploring more possible mechanism of anti-tumor activity in mice immunized with hDKK1-hHSP70 fusion vaccine. There was a significant increase in the percentage of CD4+IFN-γ+ (7.19%), compared with other groups (2.52%, 2.49%, 3.19%, 4.32%, 5.14%, 6.01%, respectively. *P* < 0.05), and in the percentage of CD8+IFN-γ+ (12.7%) compared with other groups (4.01%, 4.09%, 6.05%, 9.19%, 10.6%, 11.3%, respectively. *P* < 0.05), (Figures 7-8). In addition, we further performed the percentage of CD4+CD25+FoxP3+ by flow cytometry to understand the molecular mechanisms of regulatory T cells for tumor growth. The levels of CD4+CD25+Foxp3+ regulatory T cells were the highest in the splenocytes from the NS and Vector groups and dramatically decreased in hDKK1-hHSP70 group, there were statistically significant (*p* < 0.05).(Figure 9).

### Xenogeneic hDKK1-hHSP70 fusion vaccine increased expression of IL-4 and INF-γ *in vivo*

**Figure 10:**
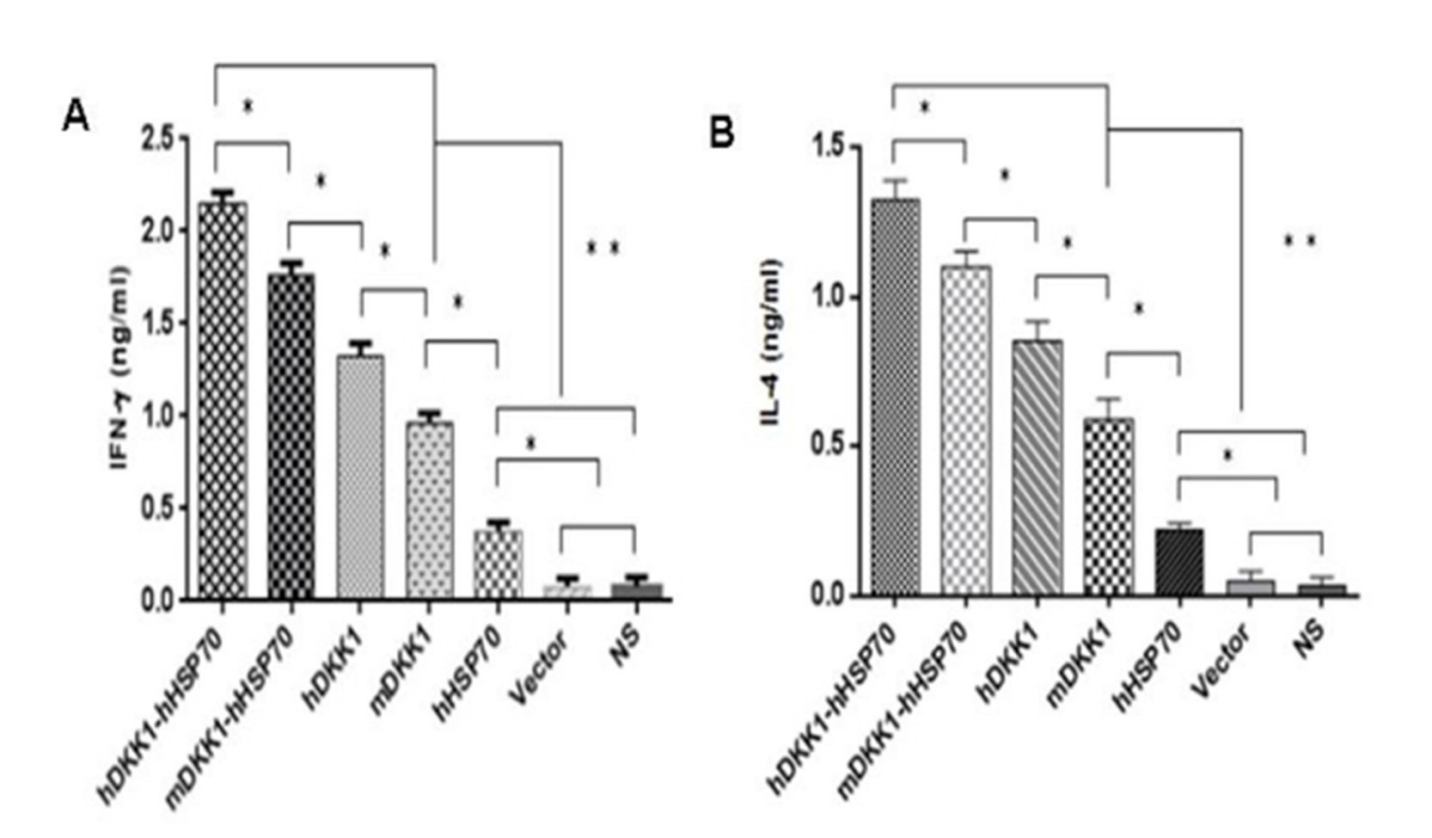
Detection of secreting IFN-γ / IL-4 of the splenocytes by ELISA A. in figure 10 is the levels of INF-γ; B. in figure 10 stand for the levels of IL-4.

We were detected the INF-γ (Th1) and IL-4 (Th2) levels in spleen lymphocytes by ELISA assay in order to further determined cell-mediated immune responses against mice multiple myeloma inoculated NS-1. The mean number of INF-γ/IL-4-secreting cells was lower levels in NS, vector and hHSP70 vaccine groups. In contrast, mice vaccination with mDKK1, hDKK1, mDKK1-hHSP70, hDKK1-hHSP70 groups had elevated levels of INF-γ/IL-4 secreting cells. Of these four, the mice vaccination with hDKK1-hHSP70 fusion DNA vaccine produced the highest levels of INF-γ/IL-4 levels. (Figure 10A and 10B).

### Xenogeneic hDKK1-hHSP70 fusion vaccine increases antigen-specific antibody immune response

**Figure 11:**
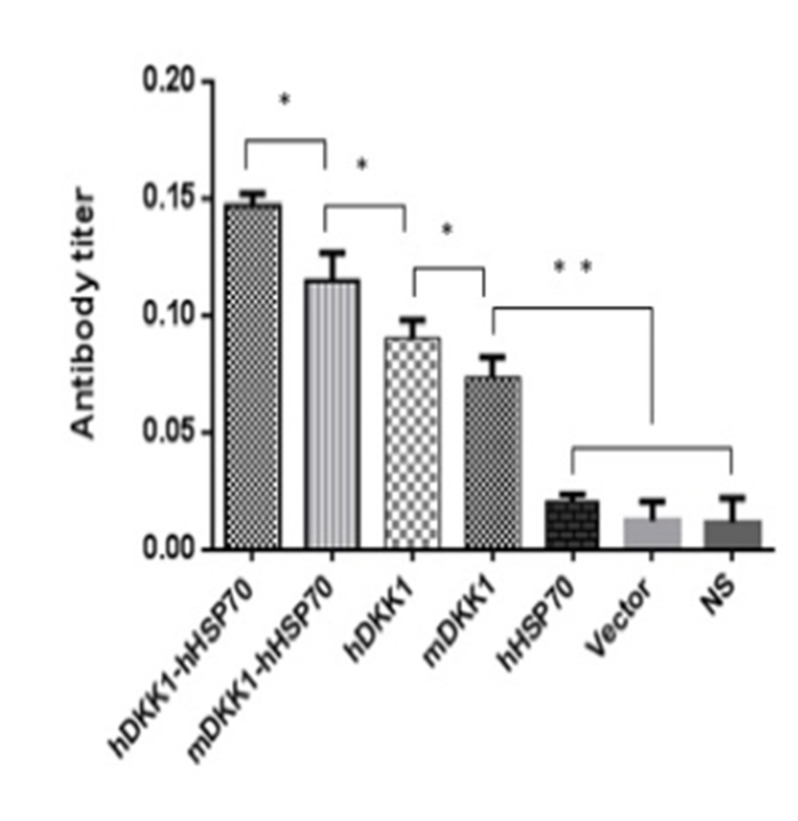
Detection of DKK1 antibody in murine serum The DKK1 antibody levels were detected by ELISA in the serum of mice one week after the third immunization. The levels of including four including DKK1 vaccine groups were higher than that of NS, Vector and hHSP70 groups (*P* < 0.05), and hDKK1-hHSP70 fusion vaccine group was highest (*P* < 0.05).*P < 0.05, ** *P* < 0.01.

We analyzed sera from naive or treated mice using ELISA for assessing the level of DKK1 antibody one week after the third immunization. There were low or undetectable levels of DKK1 antibody in sera of NS, vector, hHSP70 groups. However, DKK1-immunized mice developed significantly higher levels of DKK1 antibody than control groups (*P* < 0.05) (Figure 11). DKK1 antibody titers in the hDKK1-hHSP70 group were the highest among the groups (*P* < 0.05), (Figure 11). These results suggested that xenogeneic hDKK1-hHSP70 fusion vaccine resulted in an elevated humoral immune response against DKK1 in murine multiple myeloma models.

### Xenogeneic DKK1-HSP70 fusion vaccine effectively decreased proliferation and increased apoptosis *in vivo*

**Figure 12:**
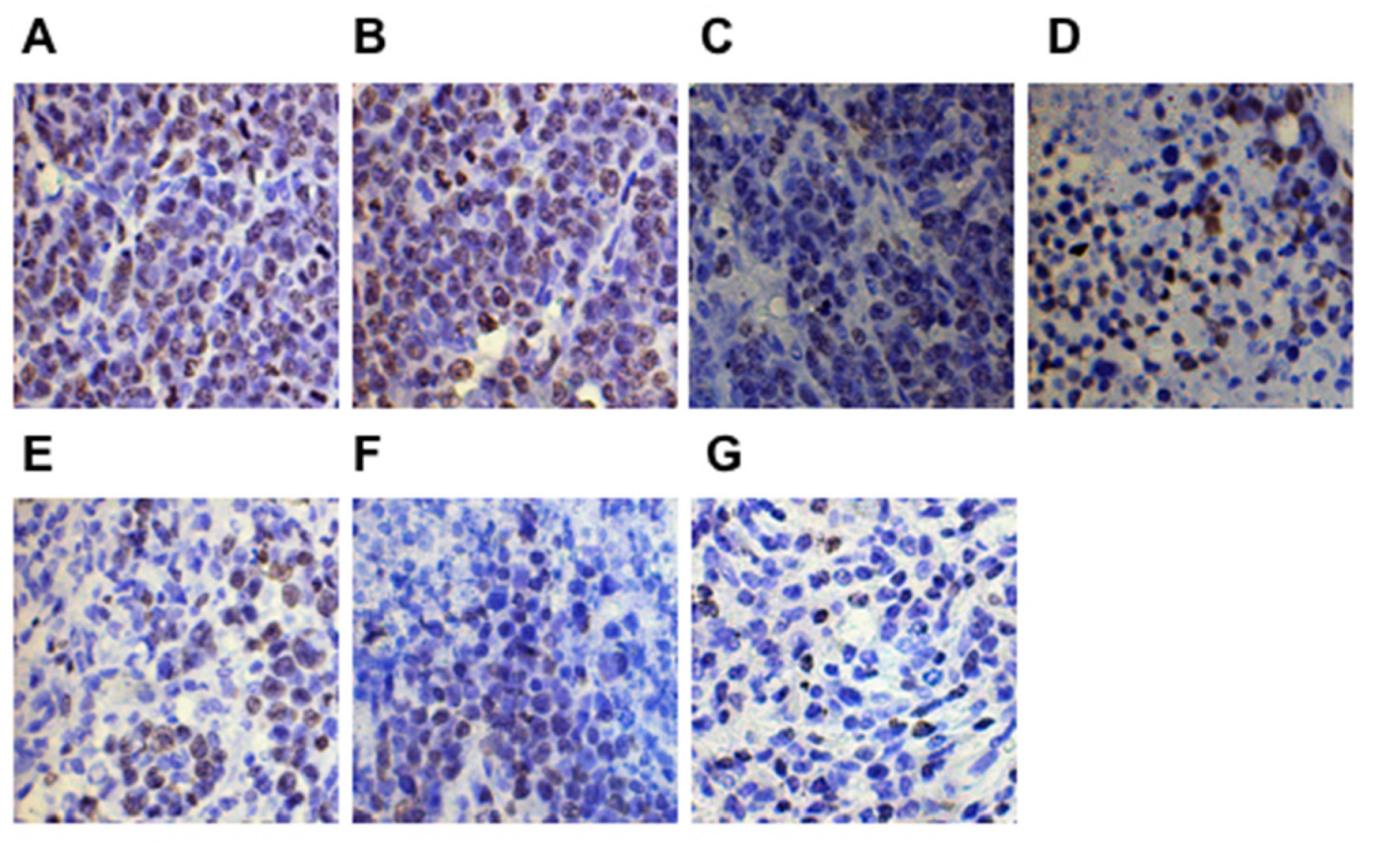
Ki-67 expression detected in tumor tissues by immunohistochemical staining (×400) The expression of Ki-67 was in cell nucleus. **A.** NS; **B.**Vector; **C.** hHSP70; **D.** mDKK1; **E.** hDKK1; **F.** mDKK1-hHSP70; **G.** hDKK1- hHSP70.

**Figure 13:**
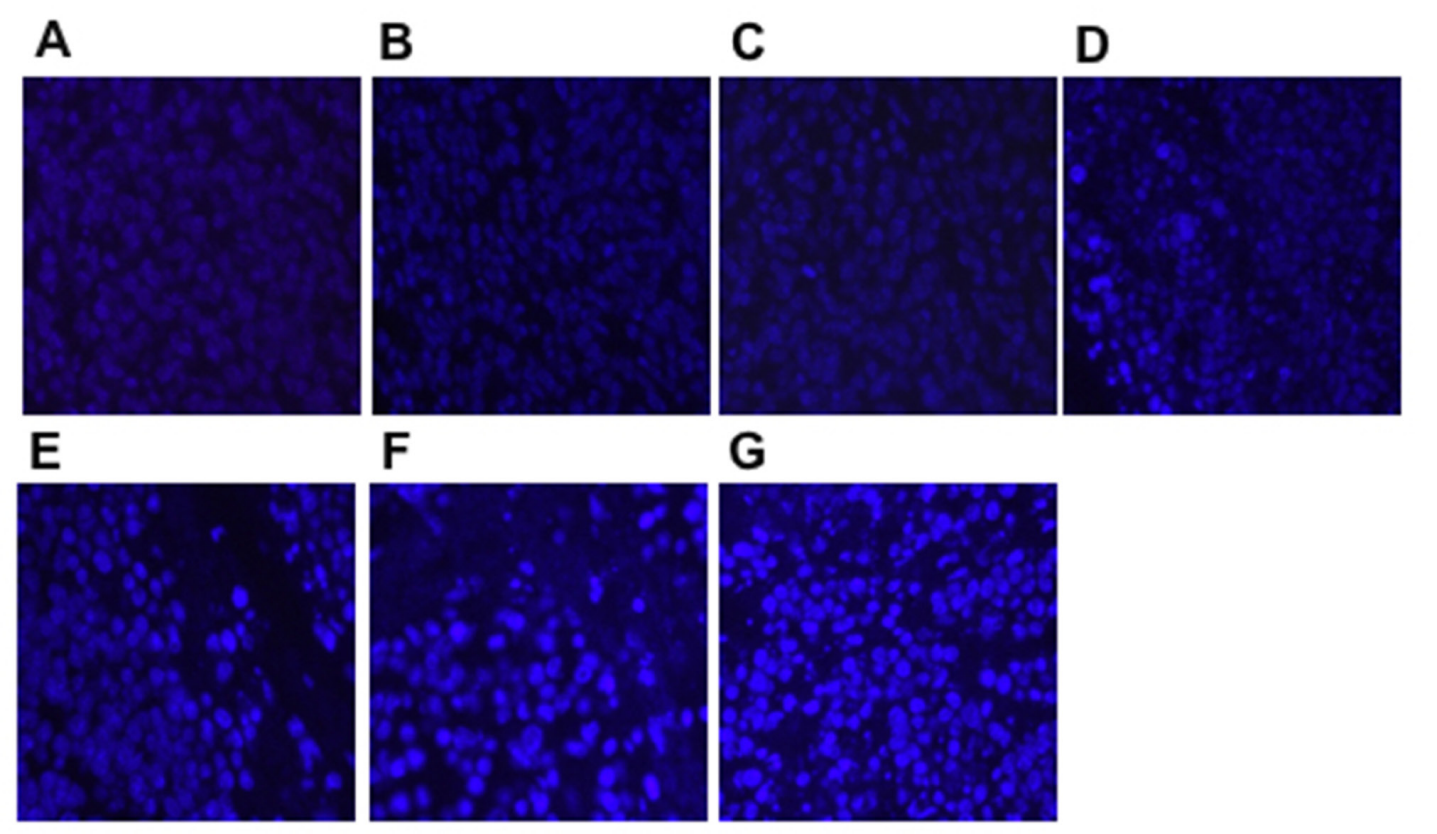
Apoptosis in tumor tissues detected by Hochest staining (×400) The density of the bright blue fluorescence was positive. **A.** NS; **B.** Vector; **C.** hHSP70; **D.** mDKK1; **E.** hDKK1; **F.** mDKK1-hHSP70; **G.** hDKK1- hHSP70.

The proliferation and apoptosis of tumors was analyzed after the last tumor volume measurement. Tumor cell proliferation was evaluated using Ki-67 staining. The expression of Ki-67 on the xenogeneic hDKK1-hHSP70 fusion vaccine-treated group was lowest (Figure 12, *P* < 0.05, *n* = 10). We also detected the apoptosis in tumor tissues by hochest staining. An apparent increase in the number of apoptotic cells was observed within the tumors from hDKK1-hHSP70 vaccine group, compared with other groups (Figure 13, *P* < 0.05, *n* = 10).

## DISCUSSION

MM remains still an incurable malignancy. As a result, we need to explore novel therapeutics to improve the outcome of patients with MM [3]. In general, MM is one of the diseases appropriated for immunotherapy because the proliferation of its cells are relatively slower compared to other hematologic malignancies such as leukemic cells [22]. Anti-tumor vaccines are considered to be one of important immunotherapeutic approaches that can overcome chemotherapy resistance and eliminate selectively malignant cells [23]. It is crucial for preparation of anti-tumor vaccines to identify tumor specific antigens (TSAs) broadly expressed in myeloma cells but highly restricted in normal tissues. In the perform study, Qian et al demonstrated that DKK1 gene and protein were expressed in most myeloma cell lines and primary myeloma cells not in normal cells, suggesting that DKK1 might be an excellent candidate as a TSA in MM [11]. In our study, we determinated that DKK1 protein was expressed in NS-1 mouse multiple myeloma.

DKK1 plays an important roles in osteolytic bone disease of MM [24]. Qian et al showed that DKK1 vaccine could protect and treat murine multiple myeloma [12]. However, generally, the induced immune response was increased after the immune adjuvants was added [25]. HSP70 has been regarded as the useful and effective anti-tumor immunoadjuvant, and a series of studies have shown that fusion vaccine including HSP70 could improve anti-tumor immune responses [11-22]. In addition, whether in prophylactic settings or therapeutic settings against cancer in rodent models, xenogeneic vaccination has stronger immunological activity than that of vaccination with ‘self’ antigens [26]. Phase I/II human clinical trials of several xenogeneic vaccines have proved that they could induce detectable immune responses against human tumor antigens and they were safe, reliable, well tolerated [26].

Based on the theoretical basis, in the present study, we constructed the mDKK1, hDKK1, mDKK1-hHSP70, hDKK1-hHSP70 DNA vaccines. We found that immunized mice with including DKK1 vaccine groups were longer median survival and was slower tumor growth than that of control groups (NS, vector, hHSP70 groups). Moreover, the survival fraction of tumor-bearing mice was the longest and the tumor growth was the slowest in hDKK1-hHSP70 fusion DNA vaccine group. These results suggested that xenogeneic hDKK1-hHSP70 fusion vaccine may effectively inhibit tumor cells *in vivo*.

To investigate the humoral immune responses, we analyzed the DKK1 antibody levels by ELISA in serum from immunized mice. In control groups, DKK1 antibody levels were low or undetectable. However, DKK1-immunized mice developed significantly higher levels of DKK1 antibody than that of control groups. In addition, DKK1 antibody titers in the hDKK1-hHSP70 group were the highest among the groups. It suggested that that xenogeneic hDKK1-hHSP70 fusion vaccine stimulated the elevated humoral immune responses against murine multiple myeloma.

The cellular immune responses were confirmed by NS-1-DKK1-specific CTLs from splenocytes of immunized mice, the percentage of CD4+IFN-γ+ and CD8+ IFN-γ+ cells and the percentage of CD4+CD25+Foxp3+ regulatory T cells. Compared with control groups, vaccination with including DKK1 vaccines groups (mDKK1, hDKK1, mDKK1-hHSP70, hDKK1-hHSP70) increased CTL cytotoxicity and DKK1-specific IFN-γ production in CD4+ cells and CD8+ cells. Moreover, hDKK1-hHSP70 fusion vaccine induced a more dramatic response than that of others DKK1 vaccines. Our experiment results also showed that hDKK1-hHSP70 vaccination could significantly decrease the percentage of CD4+CD25+Foxp3+ regulatory T cells in tumor-bearing mice. The results suggested that xenogeneic hDKK1-hHSP70 fusion vaccine initiated the cellular immune responses against murine multiple myeloma.

Our study also showed that the hDKK1-hHSP70 fusion vaccine could decrease the expression of Ki-67, increase the number of apoptosis cells. Tian et al also observed that anti-tumor vaccine of co-expressing GM-CSF and IL-18 significantly reduced the proliferation and promoted the apoptosis [27]. However, the mechanisms of anti-tumor cells proliferation and apoptosis remain unknown and need to be confirmed.

The theoretical advantages involved in this strong effect of hDKK1-hHSP70 fusion vaccine over that of others DKK1 vaccines may be mainly due to the following reasons. First, the hHSP70 fusion vaccination can significantly enhance the integrated immune response of professional antigen-presenting cells (APCs), and activate cross-present chaperoned antigenic peptides to MHC class I molecules to generate specific cytotoxic T-lymphocytes [25, 28]. Second, it can regulates the activity of DC, stimulates DC maturation and improves cross-priming of T cells mediated by DCs [29]. Third, fusion vaccination leads to the presentation of epitopes that can be presented by a variety of HLA alleles, and therefore this type of vaccine should be applicable to any patient regardless of HLA restriction [25]. In addition, the hHSP70 fusion vaccine could continuously increase the number of tumor antigens and effectively activate the immune response of specific T cells. It is more likely that build a bridge between autoimmunity and acquired immunity, which mobilize fully the body’s immune system to attack the tumor cells [30, 31].

Most of all, the antigen-specific cross-reactivity from xenogeneic homologous protein could induce stronger anti-tumor cellular responses mediated by T lymphocytes [32-34]. It is difficult for self-antigens to stimulate the immune response because of immune tolerance acquired during the development of immune system [34-36]. Therefore, the studies demonstrated that xenogeneic vaccination was more efficient than vaccination with ‘self’ antigens in rodent models in prophylactic and therapeutic settings against cancer [34]. Phase I/II human clinical trials of several xenogeneic vaccines have shown that they were safe, effective, well tolerated [26]. Consistent with these, our finding indicated that xenogeneic hDKK1-hHSP70 fusion vaccine have more stronger than immune response that of mDKK1-hHSP70 vaccine.

Meanwhile, the safety of vaccines is another important consideration in the preparation of tumor vaccines. Therefore, we also detected whether the induction of DKK1-specific immune responses was accompanied by toxicity in this study. This results suggested that no marked side effects of autoimmunity were observed in the immunized mice.

In conclusion, our experiment showed that hDKK1-hHSP70 fusion vaccine was more proficient at inhibiting tumorigenesis and progression in murine multiple myeloma. The results in this study suggested that xenogeneic hDKK1-hHSP70 vaccination may provide an effective and safety immonutheraphy strategy for multiple myeloma.

## MATERIALS AND METHODS

### Cell lines

NS-1 murine multiple myeloma cells and HEK293T cells were purchased from American Type Culture Collection (ATCC), cultured in DMEM medium with 10% FBS, and incubated in a 37°C incubator with a humidified 5% CO2 atmosphere.

### Mice

Animal studies were approved by the Ethics Review Committee for Animal Experimentation of Sichuan University. Female Balb/c mice (6-8 weeks old) were purchased from WeiTongLiHua Biological Technology Company (Beijing, China) and maintained at the Animal Center of State Key Laboratory of Biotherapy of Sichuan University.

### Vaccines preparing

The mDKK1, hDKK1, hHSP70, mDKK1-hHSP70, hDKK1-hHSP70 were synthesized through GenScript Corporation (Shanghai, China) based on the database. The mDKK1- hHSP70 and hDKK1-hHSP70 fusion cDNAs were synthesized through a linker (T2A sequence) after elimination of the stop codon of the first cDNA. All the above five cDNAs (mDKK1, hDKK1, hHSP70, mDKK1-hHSP70 and hDKK1- hHSP70) were cloned into the expression Vector, pcDNA3.1 (+), which were named as pcDNA3.1-mDKK1, pcDNA3.1-hDKK1, pcDNA3.1-hHSP70, pcDNA3.1-mDKK1-hHSP70 and pcDNA3.1-hDKK1-hHSP70, respectively. All of these plasmids were confirmed correct by diagnostic digestion and sequencing. And then, HEK293T cells were transfected with the five recombinant plasmids by liposome (Lipofectamine 2000). The cells were lysed and total cellular proteins were prepared by RIPA solution. The expression of the genes was confirmed by Western blot.

### Western blot analysis

DKK1 protein expression in NS-1 mice myeloma cells were detected by western blot analysis. Total cellular proteins were extracted from the NS-1 cells lysates and subjected to sodium dodecyl sulfate-polyacrylamide gel electrophoresis analysis. After transfer to nitrocellulose membrane and subsequent blocking, the membranes were immunoblotted with rabbit anti-mice DKK1 antibody (Santa Cruz Biotechnology, SantaCruz, CA, USA) and visualized with horseradish peroxidase (HRP)-conjugated goat anti-rabbit IgG antibody (Santa Cruz Biotechnology, SantaCruz, CA, USA), followed by enhanced chemiluminescence (Pierce Biotechnology, Rockford, IL, USA) and auto-radiography.

### Vaccination of mice

The pure plasmid DNAs were used to immunize animals by muscular injection (IM) that were divided into the prophylactic and therapeutic murine models. Each model was composed of seven groups as follows: normal saline (NS), pcDNA3.1 (Vector), pcDNA3.1-hHSP70 (hHSP70), pcDNA3.1-mDKK1 (mDKK1), pcDNA3.1- hDKK1 (hDKK1), pcDNA3.1-mDKK1-hHSP70 (mDKK1-hHSP70), pcDNA3.1- hDKK1-hHSP70 (hDKK1-hHSP70). 140 female BALB/c mice of 6-8 weeks old were randomly assigned to each group (10 mice / per group). In the prophylactic murine model, the mice were injected the plasmid DNAs (50 μg) by IM before NS-1 murine MM cells (2×10^6^ cells suspended in 100 μl of PBS / per mouse) subcutaneous injection, while in the therapeutic murine model the injection sequence was just reversed.

### Detection of DKK1 antibody

Seven days after the third immunization, serum of mice was collected, and then DKK1 specific antibody was detected using ELISA according to the manufacturer’s instructions (FangChen Corporation, Beijing, China). The absorbance of each well at 450 nm was read by the microplate reader (iMark, Bio-Rad, American). All experiments were done in triplicates.

### Cytotoxicity assays

NS-1-specific cytotoxicity mediated by cytotoxic T lymphocytes (CTLs) were assessed using the standards lactate dehydrogenase (LDH) method according to the protocal (GMS 10073 V. A, GENMED, American). The mice, seven days after the final immunization, were sacrificed and splenocytes were isolated as effector cells, and the NS-1 mouse myeloma cells were regarded as the target cells. NS-1 target cells were plated at 1×104 cells / well on 96-well U-bottomed plates, and the splenocytes (effectors) were added to a final volume of 100 μl in ratios of 1:12.5, 1:25, and 1:50 (target to effector). The plates were then incubated for 4 hours in a humidified 5% CO2 chamber at 37°C and centrifuged at 250 g for 10 min. Half of volume (50 μl) were moved from all wells to fresh 96-well flat-bottom plates, and an equal volume of reconstituted substrate mix was added per well. The plates were then incubated in the dark at room temperature for 30 min, then added the 50 μl stop solution, and finally measured the absorbance at 490 nm. The cell death percentages at each effector-to-target cell ratio were calculated using the following formula: (A490nm (experimental) − A490nm (effector spontaneous) − A490nm (target spontaneous)) / (A490nm (target maximum) - A490nm (effector spontaneous) − A490nm (target spontaneous)) × 100.

### Flow cytometry

The level of CD4+IFN-γ+ and CD8+ IFN-γ+ cells and the level of CD4+ CD25+ Foxp3+ regulatory T cells were detected by flow cytometry using anti-mouse antibodies of IFN-γ-PE, CD8-FITC, CD4-APC, CD25-PE, Foxp3-FITC (BD Bioscience Pharmingen, USA) according to the manufacturer’s instructions. Briefly, splenocytes were isolated and suspended in PBS one week after the last immunization, and then they were incubated with above mentioned antibodies at room temperature for 30 min. At last, the cells were analyzed by a BD FACS Calibur (Becton Dickinson, Franklin Lakes, NJ, USA ).

### ELISA

One week after the final immunization, the immunized mice were sacrificed and the splenocytes were collected, and then ELISA assays were carried out using the mouse IFN-γ /IL-4 ELISA kit (Dakewe Biotech Company, Shenzheng, China) according to the manufacturer’s instructions. Briefly, 5×105 splenocytes were isolated and then stimulated with NS-1 cell lysis buffer including DKK1 protein in 100 μl / well RPMI 1640 supplemented with 10% FBS at 37°C for 48 h. The supernatants were collected and serial diluted standards and samples were seeded into 96-well plate. After adding reagents, washing, chromogenic development and quenching as described in the manual instruction of test kit. The absorbance value at 450 nm was measured for each well. A linear regression was performed based on the standards for further deducing the concentration of test samples.

### HE staining and immunohistochemistry

All samples including tumor specimens, as well as heart, liver, spleen lung and kidney tissues were fixed in 4% paraformaldehyde at room temperature and subsequently dehydrated in graded ethanol, embedded in paraffin and then cut into 5μm sections for HE staining and IHC.

The sections were deparaffinized with xylene and rehydrated through graded alcohol solutions. Antigen retrieval was performed for 2 min at 120°C in citrate buffer (10mmol / L,PH 6.0) in a pressure cooker. The sections were incubated at room temperature in 0.2% hydrogen peroxide for 20 min to block the action of endogenous peroxidase, and then were separately incubated with anti-Ki-67 mAbs (1:100 dilution; Abcam, Cambridge, MA) overnight at 4°C. Subsequently, the sections were incubated with biotinylated anti-mouse antibody and streptavidin-biotinylated peroxidase complex. Finally, all sections were developed color with DAB chromogen, counterstained with hematoxylin, dehydrated, hyalinized, sealed with neutral gun, and observed under a light microscope. For each site, three pathologists performed a blind read of the glass slides.

### Hochest 33258 staining

The tumor tissues were stained with Hochest 33258 (BiYunTian Corporation, China) to evaluate the apoptotic cells. Briefly, the paraffin specimens were sliced into 5μm and stained with 33258, and observed under the fluorescence microscope (Olympus, Tokyo, Japan). The cells with highly condensed and brightly staining nuclei were regarded as apoptosis cells. The apoptotic index was defined as the ratio of the apoptotic cell number to total cell number.

### Tumor burdens

Tumor burdens were monitored by length and width when they became palpable. The tumor volume (in mm3) was calculated by the following formula: 0.5 x length (mm) x width (mm 2). At the same time, the murine survival was monitored and plotted as Kaplan-Meier curves.

### Monitoring the side effects of vaccines

The heart, liver, spleen, lung, and kidney of mice were collected, embedded and sectioned for histological examinations when the animals sacrificed. The body weight, appetite, hair and activity of mice were also observed.

### Statistical analysis

One-way analysis of variance (ANOVA) followed by Tukey’s multiple-range testing were carried out to compare the data from multiple groups using using the Graphpad Prism6 statistical software (Corporation). All values are expressed as the means ± SD. *P* < 0.05 was considered to indicate a statistically significant difference.

## References

[1] Krstevska SB, Sotirova T, Balkanov T, Genadieva-Stavric S (2014). Tretatment approach of nontransplant patients with multiple myeloma. Mater Sociomed.

[2] Munshi NC, Anderson KC (2013). New strategies in the treatment of multiple myeloma. Clin Cancer Res.

[3] Zhang L, Gotz M, Hofmann S, Greiner J (2012). Immunogenic targets for specific immunotherapy in multiple myeloma. Clin Dev Immunol.

[4] Rosenberg PS, Barker KA, Anderson WF (2015). Future distribution of multiple myeloma in the United States by sex, age, and race/ethnicity. Blood.

[5] Anderson KC, Carrasco RD (2011). Pathogenesis of myeloma. Annu Rev Pathol.

[6] Yi Q (2009). Novel immunotherapies. Cancer J.

[7] Kristinsson SY, Landgren O, Dickman PW, Derolf AR, Bjorkholm M (2007). Patterns of survival in multiple myeloma: a population-based study of patients diagnosed in Sweden from 1973 to 2003. J Clin Oncol.

[8] Sharma P, Wagner K, Wolchok JD, Allison JP (2011). Novel cancer immunotherapy agents with survival benefit: recent successes and next steps. Nat Rev Cancer.

[9] Mellman I, Coukos G, Dranoff G (2011). Cancer immunotherapy comes of age. Nature.

[10] Zhou F, Meng S, Song H, Claret FX (2013). Dickkopf-1 is a key regulator of myeloma bone disease: opportunities and challenges for therapeutic intervention. Blood Rev.

[11] Qian J, Xie J, Hong S, Yang J, Zhang L, Han X, Wang M, Zhan F, Shaughnessy JD, Epstein J, Kwak LW, Yi Q (2007). Dickkopf-1 (DKK1) is a widely expressed and potent tumor-associated antigen in multiple myeloma. Blood.

[12] Qian J, Zheng Y, Zheng C, Wang L, Qin H, Hong S, Li H, Lu Y, He J, Yang J, Neelapu S, Kwak LW, Hou J, Yi Q (2012). Active vaccination with Dickkopf-1 induces protective and therapeutic antitumor immunity in murine multiple myeloma. Blood.

[13] Wei YQ, Wang QR, Zhao X, Yang L, Tian L, Lu Y, Kang B, Lu CJ, Huang MJ, Lou YY, Xiao F, He QM, Shu JM (2000). Immunotherapy of tumors with xenogeneic endothelial cells as a vaccine. Nat Med.

[14] Weber LW, Bowne WB, Wolchok JD, Srinivasan R, Qin J, Moroi Y, Clynes R, Song P, Lewis JJ, Houghton AN (1998). Tumor immunity and autoimmunity induced by immunization with homologous DNA. J Clin Invest.

[15] Steitz J, Bruck J, Steinbrink K, Enk A, Knop J, Tuting T (2000). Genetic immunization of mice with human tyrosinase-related protein 2: implications for the immunotherapy of melanoma. Int J Cancer.

[16] Seledtsov VI, Goncharov AG, Seledtsova GV (2015). Multiple-purpose immunotherapy for cancer. Biomed Pharmacother.

[17] Wang L, Rollins L, Gu Q, Chen SY, Huang XF (2009). A Mage3/Heat Shock Protein70 DNA vaccine induces both innate and adaptive immune responses for the antitumor activity. Vaccine.

[18] Feder ME, Hofmann GE (1999). Heat-shock proteins, molecular chaperones, and the stress response: evolutionary and ecological physiology. Annu Rev Physiol.

[19] Chen DX, Su YR, Shao GZ, Qian ZC (2004). Purification of heat shock protein 70-associated tumor peptides and their antitumor immunity to hepatoma in mice. World J Gastroenterol.

[20] Binder RJ (2006). Heat shock protein vaccines: from bench to bedside. Int Rev Immunol.

[21] Zong J, Peng Q, Wang Q, Zhang T, Fan D, Xu X (2009). Human HSP70 and modified HPV16 E7 fusion DNA vaccine induces enhanced specific CD8+ T cell responses and anti-tumor effects. Oncol Rep.

[22] Weng Y, Shao L, Ouyang H, Liu Y, Yao J, Yang H, Luo Y, Wang H, Zhao Z, Mou H, Zhou Z, Zhang Y (2012). A unique MUC1-2-VNTR DNA vaccine suppresses tumor growth and prolongs survival in a murine multiple myeloma model. Oncol Rep.

[23] Danylesko I, Beider K, Shimoni A, Nagler A (2012). Novel strategies for immunotherapy in multiple myeloma: previous experience and future directions. Clin Dev Immunol.

[24] Menezes ME, Devine DJ, Shevde LA, Samant RS (2012). Dickkopf1: a tumor suppressor or metastasis promoter?. Int J Cancer.

[25] Jiang J, Xie D, Zhang W, Xiao G, Wen J (2013). Fusion of Hsp70 to Mage-a1 enhances the potency of vaccine-specific immune responses. J Transl Med.

[26] Cavallo F, Aurisicchio L, Mancini R, Ciliberto G (2014). Xenogene vaccination in the therapy of cancer. Expert Opin Biol Ther.

[27] Tian H, Shi G, Yang G, Zhang J, Li Y, Du T, Wang J, Xu F, Cheng L, Zhang X, Dai L, Chen X, Zhang S (2014). Cellular immunotherapy using irradiated lung cancer cell vaccine co-expressing GM-CSF and IL-18 can induce significant antitumor effects. BMC Cancer.

[28] Bendz H, Ruhland SC, Pandya MJ, Hainzl O, Riegelsberger S, Brauchle C, Mayer MP, Buchner J, Issels RD, Noessner E (2007). Human heat shock protein 70 enhances tumor antigen presentation through complex formation and intracellular antigen delivery without innate immune signaling. J Biol Chem.

[29] Yuan J, Kashiwagi S, Reeves P, Nezivar J, Yang Y, Arrifin NH, Nguyen M, Jean-Mary G, Tong X, Uppal P, Korochkina S, Forbes B, Chen T (2014). A novel mycobacterial Hsp70-containing fusion protein targeting mesothelin augments antitumor immunity and prolongs survival in murine models of ovarian cancer and mesothelioma. J Hematol Oncol.

[30] Calderwood SK, Gong J, Stevenson MA, Murshid A (2013). Cellular and molecular chaperone fusion vaccines: targeting resistant cancer cell populations. Int J Hyperthermia.

[31] Joly AL, Wettstein G, Mignot G, Ghiringhelli F, Garrido C (2010). Dual role of heat shock proteins as regulators of apoptosis and innate immunity. J Innate Immun.

[32] Monzavi-Karbassi B, Luo P, Jousheghany F, Torres-Quinones M, Cunto-Amesty G, Artaud C, Kieber-Emmons T (2004). A mimic of tumor rejection antigen-associated carbohydrates mediates an antitumor cellular response. Cancer Res.

[33] Parsons T, Spendlove I, Nirula R, Writer M, Carter G, Carr F, Durrant LG (2004). A novel CEA vaccine stimulates T cell proliferation, gammaIFN secretion and CEA specific CTL responses. Vaccine.

[34] Zhang W, Liu J, Wu Y, Xiao F, Wang Y, Wang R, Yang H, Wang G, Yang J, Deng H, Li J, Wen Y, Wei Y (2008). Immunotherapy of hepatocellular carcinoma with a vaccine based on xenogeneic homologous alpha fetoprotein in mice. Biochem Biophys Res Commun.

[35] Marrack P, Kappler J, Kotzin BL (2001). Autoimmune disease: why and where it occurs. Nat Med.

[36] Boon T, Coulie PG, Van den Eynde B (1997). Tumor antigens recognized by T cells. Immunol Today.

